# Leukemia cutis with *IDH1*, *DNMT3A* and *NRAS* mutations conferring resistance to venetoclax plus 5-azacytidine in refractory AML

**DOI:** 10.1186/s40364-020-00246-9

**Published:** 2020-11-25

**Authors:** JingHan Wang, Xingnong Ye, Cuihua Fan, Jie Zhou, Shuna Luo, Jingxia Jin, Dan Chen, Yan Zheng, Cai Wu, Xiaoqiong Zhu, Jie Jin, Jian Huang

**Affiliations:** 1 Department of Hematology, The First Affiliated Hospital, Zhejiang University College of Medicine, Hangzhou, P.R. China; 2 Key Laboratory of Hematologic Malignancies, Diagnosis and Treatment, Zhejiang Hangzhou, PR China; 3 Department of Hematology of the Fourth Affiliated Hospital Zhejiang University School of Medicine, Yiwu, P.R. China; 4 Department of Hematology, Shulan Hospital, Hangzhou, P.R. China

**Keywords:** Acute myeloid leukemia, BCL-2 inhibitors, Leukemia cutis

## Abstract

Recently, novel drugs like venetoclax plus 5-azacytidine (VA) were reported to have promising efficacy in refractory acute myeloid leukemia (AML). However, there are still some cases presented with novel drugs resistance, and its genetics composition and clinical phenotype are urging to study. We described a 58-year-old patient who was resistant to intensive chemotherapy. This refractory AML was presented with the persistence of *RUNX1*, *IDH1* and *DNMT3A* mutations. *RUNX1* mutations disappeared and leukemia cutis ensued after multiple chemotherapies. Leukemia cutis exhibited *NRAS* mutations in addition to *IDH1* and *DNMT3A* mutations. With the VA salvage treatment, platelets were recovered to the normal level and blasts in bone marrow and peripheral blood were moderately controlled. However, leukemia cutis did not resolve. Unexpectedly, BM blasts obtained the new *NRAS* mutations after VA treatment, and consequently experienced leukostasis with two distinct leukemia clones. After survival of 230 days, this patient died because of spontaneous cerebral hemorrhage. This case highlights presentation of leukemia cutis with simultaneous mutations of *IDH1*, *DNMT3A* and *NRAS* in AML patients might act as a resistant niche to avoid the toxicity of multiple drugs including VA. There is unmet need to validate this result in the clinical trials or a large cohort of patients in the future.

**Supplementary Information:**

The online version contains supplementary material available at 10.1186/s40364-020-00246-9.

**To the Editor:**

Approximately 30% of newly diagnosed patients with acute myeloid leukemia (AML) do not achieve complete remission with intensive induction therapy, and therefore are classified as refractory or resistant disease (RRD) [1]. RRD is among the most challenging scenarios in AML management. With the growing clinical translation of genomics into daily routine [2–5], RRD has been becoming an important field for novel drug investigation. Recently, the well-tolerated regimens venetoclax plus 5-azacytidine (VA) were proved to be highly effective in these patients [6]. However, the features related to VA resistance are still under investigation. Here, we present with a RRD patient with a clinical and molecular picture of VA resistance.

The patient is a 58-year-old man with a morphological and immunological diagnosis of AML-M2 (Fig. 1a-b) and a past history of myocardial infarction (MI). His physical examination was unremarkable. At the time of diagnosis, the percentage of blasts was 66%, and the karyotype was normal (Figure S1). A peripheral blood test was notable for a substandently leukocytosis with WBC 104 × 10^^9^/L, hemoglobin 104 g/L, and platelets 60 × 10^^9^/L. As shown in treatment flowchart (Figure S2), induction chemotherapy with HAA based regimens (homoharringtonin 2 mg/m2 daily for 7 days, cytarabine 100 mg/m2 daily for 7 days, aclarubicin 20 mg daily for 7 days) was started [7], but bone marrow (BM) blasts reached 9% on day 15 and surged up to 36% on day 30 indicating poor response (Figure S3). NGS analyses had revealed *IDH1* (exon4:c.394C > G:p.Arg132Gly), *DNMT3A* (exon19:c.2078G > A:p.Arg693His) and *RUNX1*(exon1:c.86 T > C:p.Leu29Ser) mutations (Fig. 2 and Table S1). Based on genetic results, decitabine plus standard IA regimen (decitabine 20 mg/m2, days 1–5; idarubicin 10 mg/m2 daily for 3 days and cytarabine 100 mg/m2 daily for 7 days) were used as the re-induction therapy. About 1 month later, bone marrow smear revealed a morphological complete remission (CR) with 3% blasts, while platelet was not recovered (Figure S3B). Thus, CR with incomplete platelet recovery was rendered. Therefore, treatment with decitabine plus IA was immediatetly initiated as a bridge to allogeneic hematopoietic stem cell transplantation. Unfortunately, he began to note skin lesion, although BM blasts were stable for approximately 2 months. After treatment of 130 days, leukemia cells increased up to 20% in the peripheral blood and 6% in bone marrow with a normal karyotype (Figure S1). Physical examination showed numerous dermal gray-blue papules (Fig. 1d). No evidence of leukemia blasts involvement was observed in the Computed Tomography lung screening, hepatic ultrasound, and cerebral Magnetic Resonance Imaging, respectively (Figure S4). Biopsy of the skin lesion demonstrated a dermal infiltration of myeloblast population, which was illustrated by diffuse reactivity for CD15 and MPO immunostains (Fig. 1e-f-g). Notably, NGS demonstrated *NRAS* (exon2:c.38G > A:p.Gly13Asp)*, DNMT3A* and *IDH1* (Table S1) mutations coexisting in leukemia cutis, but *RUNX1* negativity and *DNMT3A* and *IDH1* positivity exhibiting in refractory BM samples, which was distingusished with the initially mutated pattern of BM blasts. Putting the leukemia cutis and the chemodrug resistant blasts together, AML refractory disease was definitely diagnosed. As DiNardo et al. reported using ivosidenib (an inhibitor of mutant IDH1) to treat *IDH1*-mutated relapsed or refractory AMLs, the median durations of responses were more than 8 months with 30.4% CR rate [8]. The major side effects were differentiation syndrome and prolongnation of QT interval. Based on these studies, this patient might not fit for ivosidenib treatment due to the MI history. By contrast, another novel drug venetoclax was also sensitive in *IDH1* mutant primary AML cells with less drug toxicity [9, 10]. Thus, we treated this patient with venetoclax combining with 5-azacytidine (venetoclax 400 mg and intravenous azacitidine 75 mg/m^2^ [days 1–7 of each 28-day cycle]) [6]. At venetoclax initiation, despite WBC up to 20 × 10^9/L, we did not observe tumor lysis syndrome. With this regimen, platelets were recovered to the normal level and blasts (4.5 and 6.7% respectively in bone marrow and peripheral blood) were moderately controlled. However, the patient’s skin lesion did not resolve during the course of VA treatment. At the survival time of 200 days, WBC increased rapidly up to more than 50 × 10^9/L and the immunophenotypic data revealed two clonal architecture of neoplasia in the peripheral blood (Fig. 1c). In addition, NGS diagnosis showed the same as its initial mutated genes of *IDH1* and *DNMT3A* were still positive and unexpectedly *NRAS* mutation in blood was incurred after 1 months of VA treatment (Fig. 2 and Table S1). We hypothezed the action of myeloid leukemia clones transferring to skin tissue as a resistant niche to avoid the toxicity of venetoclax. Therefore, we measured the expression of BCL-2 of both BM biopsy (Fig. 1h) and cutaneous blasts (Fig. 1i). As a result, the more and higher expression of BCL-2 was observed in BM biopsy than in leukemia cutis. After survival of 230 days, this patient unfortunately died because of spontaneous cerebral hemorrhage.

**Fig. 1:**
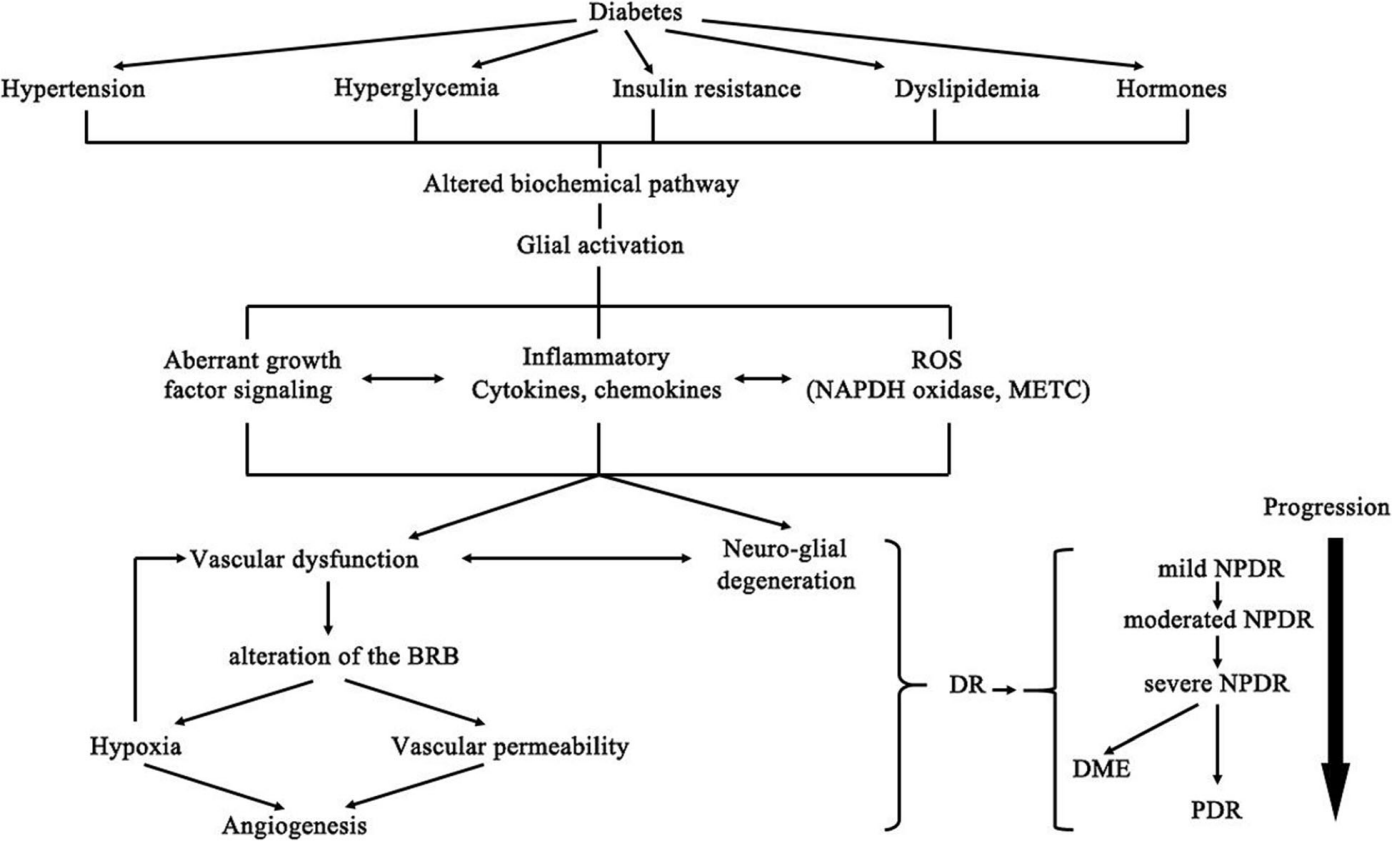
Clinical, molecular and histological features of this refractory AML patients.(A) The morphology of bone marrow (BM) blasts(× 1000). (B) The immunophenotype of BM blasts at diagnosis. Red colors in J region represent BM blasts. Flow cytometry determines 45% blasts and demonstrates MPO, CD38 and HLA-DR dim expression, CD33, CD117, CD13, CD34, CD9 and CD123 positivity, while CD2, CD5, CD7, CD10, CD19, CD79a, CD11b negativity. (C) The immunophenotype of peripheral blood cells when leukemocytosis ensues. Red and blue colors illustrate two leukemia clones. (D) Leukemia cutis in this patient. (E) The morphology analysis in skin biopsy sample (H&E × 100). (F) The immunohistochemistry displays MPO positive in skin biopsy sample (× 100). (G) The immunohistochemistry displays CD15 positive in skin biopsy sample (× 100). (H) The immunohistochemistry displays BCL-2 positive in blasts of the bone marrow biopsy (× 100). (I) The immunohistochemistry displays a morsol of BCL-2 positive in skin biopsy sample (× 100)

**Fig. 2:**
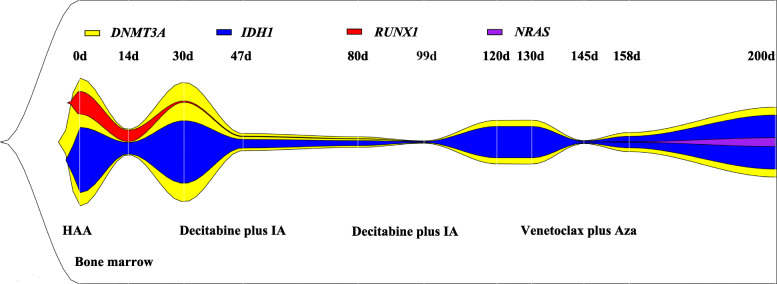
The clone progression following mulitple chemodrugs and venetoclax treatment. Schematic of a possible model clone evolution inferred from next-generation sequencing data combined with the percentage of blasts detected by flow cytometry and visualized using fishplot R packages [11]. Here, we found the variate allele frequence (VAF) of *DNMT3A* is higher than that of *IDH1* mutation at daignosis, implying *IDH1* mutations occurring later than *DNMT3A* mutations. Following the intensive chemotherapy, *RUNX1* mutated clone disappeared, but *IDH1* and *DNMT3A* mutated clones still survived. Notably, the leukeiam cutaneous with additional *NRAS* mutations did not resolve during VA regimens. Thus, these clones in skin can persist over the time of the intensive chemotherapy and obtain resistance to targeted therapy, leading to further clonal expansion and eventually causing recurrent disease in the blood and bone marrow

## Supplementary Information

**Additional file 1 Figure S1**. Normal karyotype was illustrated at the time of AML diagnosis (A), morphological complete remission (B), and leukemia refractory and resistance(C), respectively. **Figure S2**. Treatment flowchart illustrates in this study. **Figure S3**. The morphology of bone marrow (BM) blasts(× 1000) indicated no complete remission on day 30 after HAA treatment(A), a morphological complete remission after treatment with decitabine plus IA(B), leukemia relapse after the second course of decitabine plus IA(C), and moderately control under VA treatment. **Figure S4**. No evidence of leukemia blasts involvement was observed in the Computed Tomography lung screening (A), hepatic ultrasound(B), and cerebral Magnetic Resonance Imaging(C), respectively. **Table S1**. The detailed information of gene mutations.

## Abbreviations

VA: Venetoclax plus 5-azacytidine
AML: Acute myeloid leukemia
BM: Bone marrow
