# Supplementary material for: Leukemia cutis with IDH1, DNMT3A and NRAS mutations conferring resistance to venetoclax plus 5-azacytidine in refractory AML
Source: Biomark Res. 2020 Nov 25;8:65. doi: 10.1186/s40364-020-00246-9 (PMC7687845; doi:10.1186/s40364-020-00246-9)
Supplement: Supplementary file 1 — Additional file 1 Figure S1. Normal karyotype was illustrated at the time of AML diagnosis (A), morphological complete remission (B), and leukemia refractory and resistance(C), respectively. Figure S2. Treatment flowchart illustrates in this study. Figure S3. The morphology of bone marrow (BM) blasts(× 1000) indicated no complete remission on day 30 after HAA treatment(A), a morphological complete remission after treatment with decitabine plus IA(B), leukemia relapse after the second course of decitabine plus IA(C), and moderately control under VA treatment. Figure S4. No evidence of leukemia blasts involvement was observed in the Computed Tomography lung screening (A), hepatic ultrasound(B), and cerebral Magnetic Resonance Imaging(C), respectively. Table S1. The detailed information of gene mutations. [file 40364_2020_246_MOESM1_ESM.docx]

**Leukemia cutis with *IDH1*, *DNMT3A* and *NRAS* mutations conferring resistance to venetoclax plus 5-azacytidine in refractory AML**

**
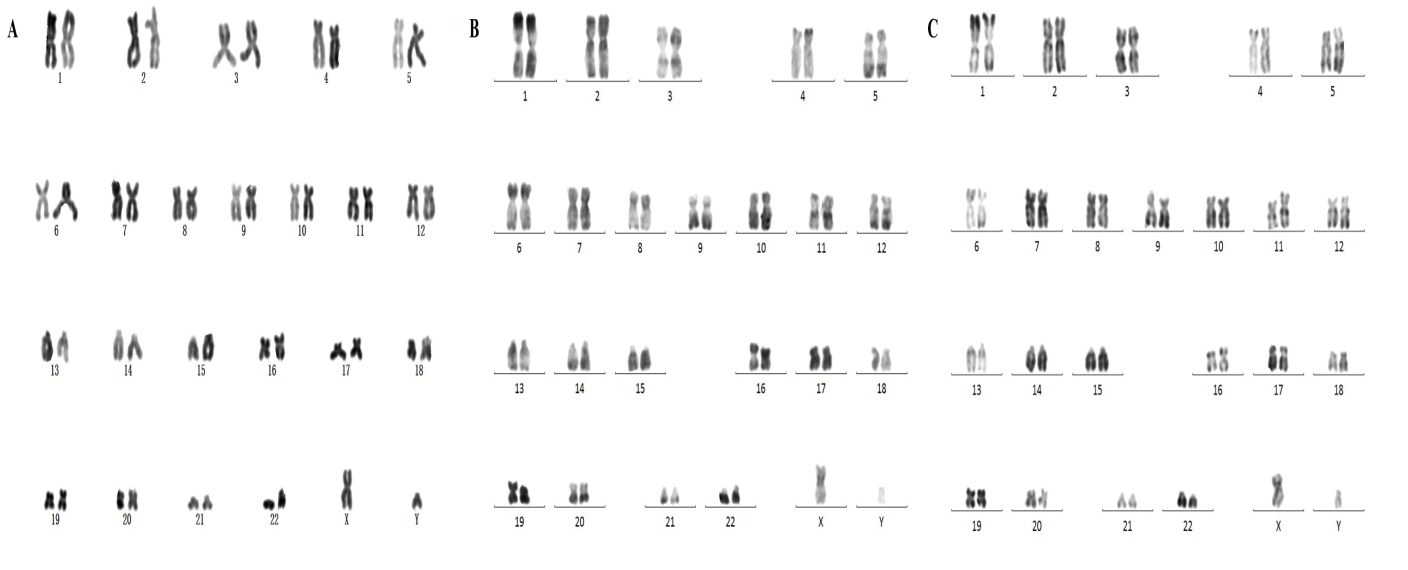
**

Figure S1. Normal karyotype was illustrated at the time of AML diagnosis (A), morphological complete remission (B), and leukemia refractory and resistance(C), respectively.


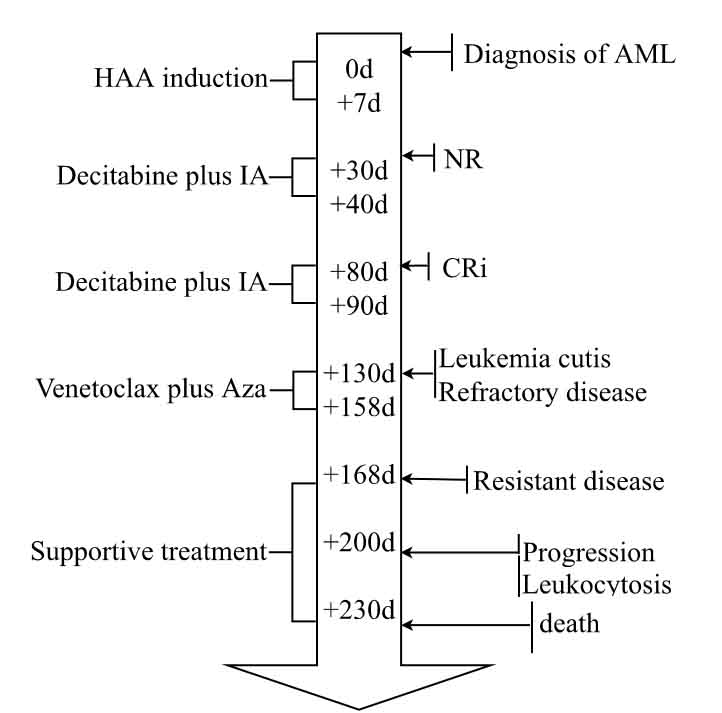


Figure S2. Treatment flowchart illustrates in this study.


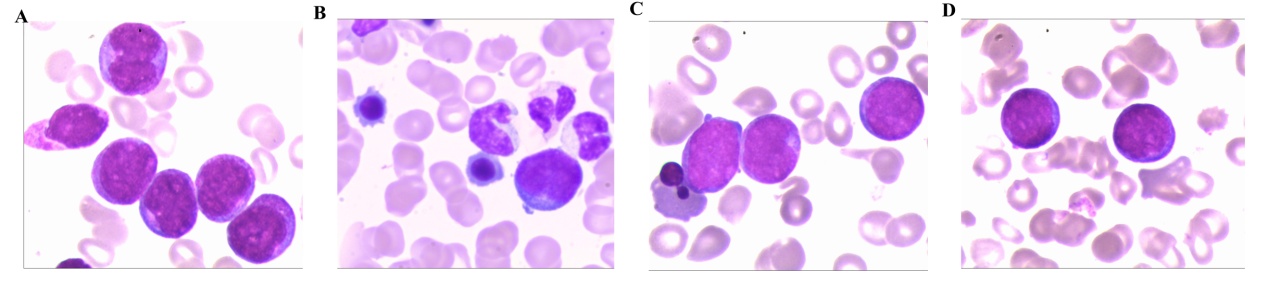


Figure S3. The morphology of bone marrow (BM) blasts(×1000) indicated no complete remission on day 30 after HAA treatment(A), a morphological complete remission after treatment with decitabine plus IA(B), leukemia relapse after the second course of decitabine plus IA(C), and moderately control under VA treatment.


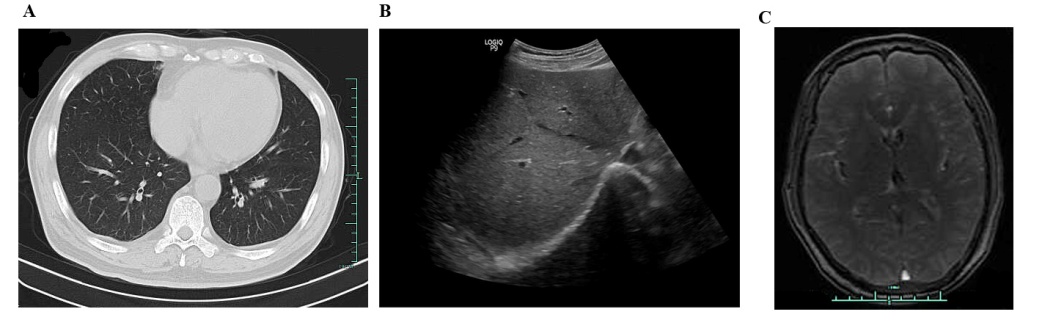


Figure S4. No evidence of leukemia blasts involvement was observed in the Computed Tomography lung screening (A), hepatic ultrasound(B), and cerebral Magnetic Resonance Imaging(C), respectively.

Table S1. The detailed information of gene mutations.

| Bone marrow samples at diagnosis | | | |  |  | |
| --- | --- | --- | --- | --- | --- | --- |
| Gene | Transcript | Position | DNA_Change | Protein_Change | Variation_Allele_Frequenc(%） |  |
| DNMT3A | NM_022552 | Exon23 | c.2645G>A | p.R882H | 48.2 |  |
| IDH1 | NM_005896 | Exon4 | c.394C>G | p.R132G | 36.4 |  |
| RUNX1 | NM_001001890 | exon1 | c.86T>C | p.Leu29Ser | 8.3 |  |
| Bone marrow samples after treatment of 130 days | | | | | |  |
| DNMT3A | NM_022552 | Exon23 | c.2645G>A | p.R882H | 46.3 |  |
| IDH1 | NM_005896 | Exon4 | c.394C>G | p.R132G | 24.6 |  |
| Blood samples after survival of 200 days | | | | |  |  |
| NRAS | NM_002524 | Exon2 | c.38G>A | p.G13D | 15.7 |  |
| DNMT3A | NM_022552 | Exon23 | c.2645G>A | p.R882H | 35.2 |  |
| IDH1 | NM_005896 | Exon4 | c.394C>G | p.R132G | 28.2 |  |
| Leukemia cutis | |  |  |  |  |  |
| NRAS | NM_002524 | Exon2 | c.38G>A | p.G13D | 29.1 |  |
| DNMT3A | NM_022552 | Exon23 | c.2645G>A | p.R882H | 53.3 |  |
| IDH1 | NM_005896 | Exon4 | c.394C>G | p.R132G | 16.4 |  |
